# Supplementary material for: Influence of Iron Addition (Alone or with Calcium) to Elements Biofortification and Antioxidants in Pholiota nameko
Source: Plants (Basel). 2021 Oct 24;10(11):2275. doi: 10.3390/plants10112275 (PMC8623578; doi:10.3390/plants10112275)
Supplement: Supplementary file 1 [file plants-10-02275-s001.zip › plants-1427874-supplementary.pdf]

## Supplementary data

Table S1. Parameters of analytical procedure

| Element | Calibration range up to mg $L^{-1}$ | Wavelength nm | Plasma view | DL mg $kg^{-1}$ |
|---|---|---|---|---|
| **Ca** | 2000 | 239,856 | axial | x |
| **Ca** | 200 | 315,887 | radial | 0,077 |
| **Fe** | 100 | 261,187 | radial | 0,067 |
| **Fe** | 1000 | 262,167 | axial | x |
| **K** | 2000 | 404,414 | axial | x |
| **K** | 100 | 769,897 | radial | 0,011 |
| **Mg** | 2000 | 279,078 | axial | x |
| **Mg** | 50 | 285,213 | radial | 0,041 |
| **Mn** | 20 | 257,610 | radial | 0,015 |
| **Na** | 1000 | 330,237 | axial | x |
| **Na** | 100 | 588,995 | axial | 0,013 |
| **P** | 1000 | 202,349 | axial | x |
| **P** | 30 | 213,618 | radial | 0,015 |
| **S** | 1000 | 182,565 | axial | x |
| **S** | 100 | 180,672 | radial | 0,020 |

x – calibration for high concentration of element

Table S2. Content [mg $kg^{-1}$] of determined elements in *Pholiota nameko* fruit bodies

| System | Ca | Fe | K | Mg | Mn | Na | P | S |
|---|---|---|---|---|---|---|---|---|
| Control | $233^{f}$ ± 11.8 | $8.61^{g}$ ± 1.19 | $11300^{bc}$ ± 621 | $440^{d}$ ± 17.8 | $6.71^{d}$ ± 0.965 | $104^{d}$ ± 4.65 | $6640^{cd}$ ± 290 | $3600^{e}$ ± 137 |
| $Fe_{5}$ | $241^{f}$ ± 19.1 | $15.9^{f}$ ± 1.78 | $13900^{ab}$ ± 679 | $651^{c}$ ± 42.2 | $13.2^{c}$ ± 1.76 | $130^{c}$ ± 6.40 | $6950^{bc}$ ± 63.8 | $4030^{bcd}$ ± 40.4 |
| $Fe_{10}$ | $219^{f}$ ± 27.1 | $19.8^{ef}$ ± 1.46 | $17200^{ab}$ ± 404 | $852^{ab}$ ± 12.9 | $12.4^{c}$ ± 1.76 | $173^{a}$ ± 4.01 | $7230^{ab}$ ± 42.4 | $4140^{a-d}$ ± 56.0 |
| $Fe_{15}$ | $225^{f}$ ± 33.1 | $25.1^{cd}$ ± 1.54 | $19100^{a}$ ± 699 | $928^{a}$ ± 35.0 | $12.2^{c}$ ± 1.09 | $183^{a}$ ± 6.19 | $7430^{a}$ ± 70.7 | $4190^{abc}$ ± 22.7 |
| $Fe_{5}$ $Ca_{5}$ | $458^{e}$ ± 28.2 | $23.1^{de}$ ± 1.21 | $7770^{c}$ ± 4790 | $614^{c}$ ± 23.2 | $14.0^{c}$ ± 1.03 | $114^{cd}$ ± 3.28 | $6410^{de}$ ± 78.8 | $4030^{bcd}$ ± 114 |
| $Fe_{10}$ $Ca_{5}$ | $698^{d}$ ± 52.6 | $24.7^{d}$ ± 0.57 | $16300^{ab}$ ± 882 | $870^{a}$ ± 65.1 | $16.6^{bc}$ ± 0.793 | $153^{b}$ ± 3.13 | $6640^{cd}$ ± 86.9 | $4270^{ab}$ ± 50.7 |
| $Fe_{15}$ $Ca_{5}$ | $931^{c}$ ± 40.4 | $31.6^{b}$ ± 1.66 | $14400^{ab}$ ± 441 | $937^{a}$ ± 26.3 | $20.6^{b}$ ± 0.966 | $179^{a}$ ± 3.62 | $6720^{cd}$ ± 26.5 | $4330^{a}$ ± 56.0 |
| $Fe_{5}$ $Ca_{10}$ | $825^{cd}$ ± 6.60 | $27.4^{bcd}$ ± 0.94 | $12700^{bc}$ ± 319 | $724^{bc}$ ± 50.4 | $15.0^{c}$ ± 1.40 | $123^{cd}$ ± 5.99 | $6250^{e}$ ± 43.7 | $3940^{cd}$ ± 40.5 |
| $Fe_{10}$ $Ca_{10}$ | $1430^{b}$ ± 83.2 | $29.5^{bc}$ ± 1.00 | $16200^{ab}$ ± 970 | $709^{bc}$ ± 26.1 | $29.5^{a}$ ± 1.32 | $153^{b}$ ± 6.80 | $6370^{de}$ ± 39.5 | $3870^{d}$ ± 58.4 |
| $Fe_{15}$ $Ca_{10}$ | $1620^{a}$ ± 27.9 | $38.3^{a}$ ± 1.09 | $14800^{ab}$ ± 1350 | $728^{bc}$ ± 33.0 | $28.2^{a}$ ± 1.04 | $175^{a}$ ± 6.71 | $6230^{e}$ ± 51.4 | $4110^{abc}$ ± 71.1 |

n=5, identical superscripts (a, b…) in columns denote no significant differences between means according to a post-hoc Tukey's HSD test at α=95% following one-way ANOVA
